# Guiding bone formation using semi‐onlay calcium phosphate implants in an ovine calvarial model

**DOI:** 10.1002/term.3288

**Published:** 2022-02-23

**Authors:** Gry Hulsart Billström, Viviana R. Lopes, Christopher Illies, Sara Gallinetti, Jonas Åberg, Håkan Engqvist, Conrado Aparicio, Sune Larsson, Lars Kihlström Burenstam Linder, Ulrik Birgersson

**Affiliations:** ^1^ Department of Medicinal Chemistry Translational Imaging Uppsala University Uppsala Sweden; ^2^ Department of Clinical Pathology Karolinska University Hospital Stockholm Sweden; ^3^ Department of Engineering Sciences Applied Materials Science Section Uppsala University Uppsala Sweden; ^4^ Faculty of Odontology International University of Catalonia Barcelona Spain; ^5^ Department of Surgical Sciences, Orthopaedics Uppsala University Uppsala Sweden; ^6^ Department of Clinical Neuroscience Neurosurgical Section Karolinska University Hospital Stockholm Sweden; ^7^ Department of Clinical Science, Intervention and Technology Division of Imaging and Technology Karolinska Institute Huddinge Sweden; ^8^ OssDsign Uppsala Sweden

**Keywords:** additive manufacturing, animal test, bioceramics, bone, regenerative mechanism

## Abstract

The restoration of cranio‐maxillofacial deformities often requires complex reconstructive surgery in a challenging anatomical region, with abnormal soft tissue structures and bony deficits. In this proof‐of‐concept, the possibility of vertical bone augmentation was explored by suspending hemispherically shaped titanium‐reinforced porous calcium phosphate (CaP) implants (*n* = 12) over the frontal bone in a sheep model (*n* = 6). The animals were euthanized after week 13 and the specimens were subject to micro‐computed tomography (μCT) and comprehensive histological analysis. Histology showed that the space between implant and the recipient bone was filled with a higher percentage of newly formed bone (NFB) versus soft tissue with a median of 53% and 47%, respectively. Similar results were obtained from the μ‐CT analysis, with a median of 56% NFB and 44% soft tissue filling the void. Noteworthy, significantly higher bone‐implant contact was found for the CaP (78%, range 14%–94%) versus the Titanium (29%, range 0%–75%) portion of the implant exposed to the surrounding bone. The histological analysis indicates that the CaP replacement by bone is driven by macrophages over time, emphasized by material‐filled macrophages found in close vicinity to the CaP with only a small number of single osteoclasts found actively remodeling the NFB. This study shows that CaP based implants can be assembled with the help of additive manufacturing to guide vertical bone formation without decortification or administration of growth factors. Furthermore, it highlights the potential disadvantage of a seamless fit between the implant and the recipient's bone.

## 1 INTRODUCTION

Cranio‐maxillofacial (CMF) malformities and deformities can be devastating for patients in terms of physical function, social stigmatization and self‐acceptance. The restoration of the esthetical and/or physical function caused by congenital, developmental, posttraumatic, or postoncologic treatment, often requires complex reconstructive surgery in a challenging anatomical region, with abnormal soft tissue structures and bony deficits. Depending on the deformity, various treatment options are available, ranging from autologous bone grafts to various synthetic materials (Kwarcinski et al., 2017; Neovius & Engstrand, 2010; Ridwan‐Pramana et al., 2015; Song et al., 2015; van de Vijfeijken et al., 2018). The use of synthetic materials allows for simplification of the operating procedure and a reduction of operative time whilst avoiding unnecessary donor site morbidity. With the advancement of 3D‐printed patient‐specific implants, an exact fit between the implant and the bony can be achieved, allowing for perfect esthetical adaptation.

Among the most explored synthetic materials for bone repair and augmentation are various calcium phosphate (CaP) cements, which are generally cured perioperatively and allowed to set directly onto the underlying bone surface and then sculpted perioperatively to achieve favorable cosmetic outcomes (Lodoso‐Torrecilla et al., 2021). The concept of using CaP with its close resemblance to bone has been around for more than a century: already in 1920, Albee and Morrison tested triple CaP in segmental radius defects in rabbits (Albee & Morrison, 1920). Since that experiment, it has been shown that, in addition to resembling bone, CaPs are osteoconductive (allowing bone growth on their surface) and occasionally osteoinductive, that is, trigger the differentiation of stem cells to osteogenic lineage, leading to bone formation even in a non‐osseous environment (Billstrom et al., 2013; Bohner & Miron, 2019). Due to their often unpredictable osteoinductive nature, calcium phosphates have been combined with various growth factors and osteoprogenitor cells (Albrektsson et al., 1981; Billstrom et al., 2013; Han et al., 2021; Hulsart‐Billstrom et al., 2011; James et al., 2016; Tannoury & An, 2014). Even though encouraging results have been obtained, limitations remain due to adverse effects, such as the risk of bone overgrowth and bone formation in unwanted areas, varying regenerative potential as well as regulatory and budgetary restrictions (Docherty‐Skogh et al., 2010; Jeong et al., 2019; Tannoury & An, 2014).

In this proof‐of‐concept, we explore the possibility of triggering vertical bone augmentation without the addition of exogenous growth factors or cells by suspending a CaP implant over the recipient frontal bone, unlike current treatment options where a seamless fit between implant and bone is preferred. The implant under study has a hemispherical shape with a concavity in the center preventing it to be in direct contact with recipient bone (i.e., semi‐onlay) rather than what is typical of an onlay graft. We hypothesize that this cavity could act as a cast to guide bone formation, allowing bone to form both from the recipient's bone and by bone apposition on the CaP. This can be facilitated by either osteoconduction or by providing osteoinductive cues or a combination thereof, causing de novo bone formation. If successful, this could be a new way of designing implants.

## 2 MATERIALS AND METHODS

### 2.1 Study design

The study is conducted in accordance with the (i) Organisation for Economic Co‐operation and Development (OECD) Good Laboratory Practice regulations, (ii) ENV/MC/CHEM (98) 17 with the United States Food and Drug Administration Good Laboratory Practice regulations, and (iii) 21 CFR 58. Further, the study adhered to the European Union (EU) directive 2010/63/EU and were performed at NAMSA medical research center (Chasse‐Sur‐Rhone, France and was authorized by Ministry of Higher Education and Research) under the number 01139.02. The housing during the follow‐up period, which was conducted using a non‐GLP but audited and approved NAMSA sub‐contractor: La Bergerie de la Combe aux Loups ‐ ISO 9001 certified provider. The use of a non‐GLP sub‐contractor was based on the rationale that the farm setting is preferred for animal housing in long‐term studies for ethical reasons. Six Blanche du Massif Central female sheep—ranging in age from 3.4 to 4 years and weighing between 59 and 70 kg—received two implants each. Areas that were located next to the implants (i.e., the adjacent bone) were used as negative controls, which received similar pre‐treatment. The animals were euthanized after week 13 and the specimens were subject to micro‐computed tomography (μCT) and comprehensive histological analysis.

### 2.2 Material

#### 2.2.1 Sample preparation and material characterization

**FIGURE 1:**
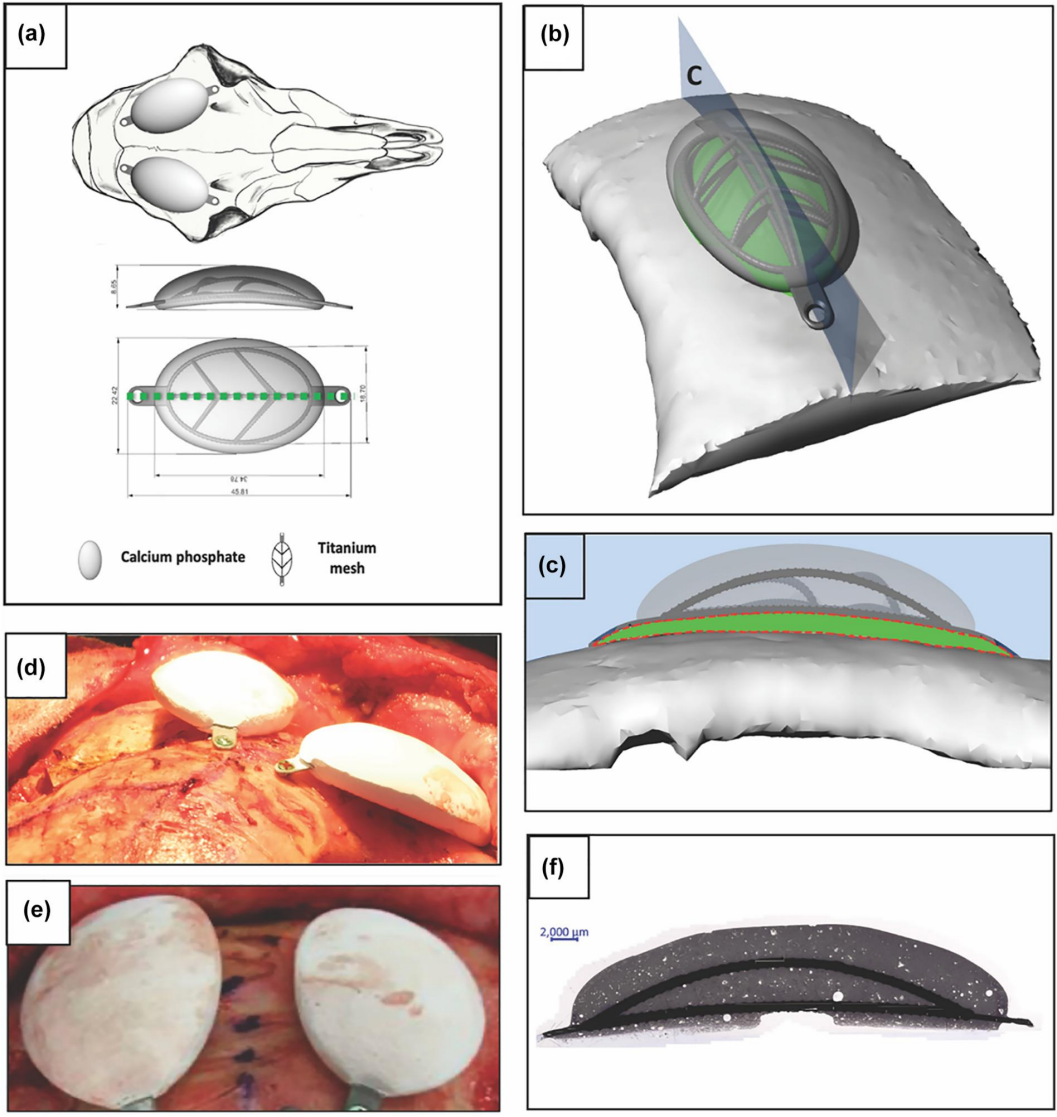
(a) An illustration of the ovine semi‐onlay model with the placement of a concave calcium phosphate (CaP) implant on the frontal part of the skull. (b) Schematic overview of the implantation on the skull showing the region of interest (ROI) for μCT in green and the histology section in blue. (c) Scheme of the histology section with the ROI for histomorphometry marked with a dotted red line. Cross‐section of μCT volumes of interest|volume of interest (VOI) seen in green. (d, e) Implant positioning at the time of implantation in two sheep. (f) Vertical cut of a native implant, in accordance with the blue section plan displayed in (b)

Twelve semi‐onlay Cranio‐maxillofacial implants (Figure 1) composed of a medical grade 1 titanium skeleton embedded in a ceramic bulk composed of Monetite, beta‐Tricalcium Phosphate (β‐TCP) and calcium pyrophosphate (CPP) were fabricated (Engstrand et al., 2014). In short, the CaP powders were mixed with glycerol (Sigma Aldrich), enabling casting under controlled conditions and setting. The cement was then shaped in a silicon cast to embed the additively manufactured titanium medical grade skeleton and was allowed to harden overnight in sterile water. After removal from the cast, the CaP implant was left in sterile water for 48 h in order to reduce the glycerol content; and finally, the samples were left to dry and sterilized at 121°C for 20 min.

#### 2.2.2 Phase composition

The phase compositions of the different calcium phosphates were ascertained through x‐ray diffraction (XRD) on 4 samples (*n* = 4). The XRD analysis was performed with an x‐ray diffractometer (Aeris Panalytical, Malvern) with a theta‐theta (2θ) setup with Ni‐filtered Cu–K irradiation. The diffraction patterns were collected with a beam knife between 2θ of 10°–60° and a step of 0.011° and 21.5 s. Further Rietveld refinements were applied to perform a quantitative phase composition analysis with HighScore Plus (Degen et al., 2014). Our crystalline models were based on literature references: CPP (Boudin et al., 1993), β‐TCP (Dickens et al., 1974) and monetite (Dickens et al., 1972). No other phases were identified in the diffraction patterns.

#### 2.2.3 Microstructure analysis

The ceramic microstructure of the bottom of the implant was visualized with scanning electron microscopy (SEM, Marvin, Zeiss, Germany). Prior to scanning, the samples were fixed with carbon tape to the sample holder, dried at 60°C and coated with Au‐Pd. The coating was sputtered at a voltage of 2 kV and a current of 15–20 mA for 60 s with a Polaron SC7640 Sputter coater (Thermo VG Scientific).

#### 2.2.4 Wettability

The wettability of the implant surface was determined by contact angle measurements of 3 samples using sessile drop and captive bubble methods. A macro contact angle meter (DM‐CE1, Kyowa, Japan) with appropriate software (FAMAS, Kyowa) was used to perform the wettability tests and calculation of contact angles. The contact angle (θ) was defined as the angle between the solid phase and liquid phase as shown in Figure 3a and b. All materials were cleaned in water and dried in a desiccator before contact angle experiments.

For the sessile method, a 2 μL drop of ultrapure water was dispensed on the surface of the tested sample (Figure 3a). With the captive bubble method, the sample was immersed in water in a small container and a bubble of air was formed underneath the surface of the tested sample (Figure 3b). In both methods, contact angles were calculated through the first 20 s of contact of the fluid with the tested surface to describe the dynamic wettability response and repeated at least three times.

### 2.3 Surgery

#### 2.3.1 Pre‐operative procedure

The animals were acclimatized for a minimum of 18 days. One day prior to surgery, animals were weighed and fasted before implantation. On the day of surgery (day 0), blood was sampled, and pre‐medication was performed by intravenous injection of a mixture of diazepam (Diazepam^®^, TVM, 0.3 mg/kg) and butorphanol (Torphasol^®^, Axience, 0.2 mg/kg). Anesthesia was induced by intravenous injection of Propofol (Propovet^®^, Axience, 2–5 mg/kg). If necessary, lidocaine was sprayed in the laryngeal area to facilitate endotracheal intubation. Each sheep was intubated, mechanically ventilated and placed on isoflurane (Isoflo^®^, Axience) for continued general anesthesia. An intravenous infusion with saline or another suitable electrolyte solution was performed during surgery. Pre‐operative intramuscular (IM) injection of an anti‐inflammatory drug (flunixin, Meflosyl^®^ Injectable, Zoetis, 2 mg/kg) and IM and/or subcutaneous injection of antibiotic treatment (amoxicillin, Duphamox^®^ LA, Zoetis, 15 mg/kg and enrofloxacin, Baytril^®^ 10%, Bayer Pharma, 5 mg/kg, respectively) was administered. A neutral ophthalmic ointment was applied to both eyes to protect the corneas from drying and was re‐applied as needed (Ocrygel^®^, Laboratoire TVM). To minimize the risk of infection, the animal preparation was done in a separate room from the surgical theater. The surgical area was shaved, scrubbed with povidone‐iodine, wiped with 70% isopropyl alcohol, painted with povidone‐iodine solution and draped. The sheep were placed in a prone position on a warming pad. A rectal temperature probe and a rumen tube were placed during surgery. Electrocardiogram (ECG), peripheral non‐invasive arterial blood pressure and oxygen saturation were monitored.

#### 2.3.2 Implantation

A midline incision was made through the skin from the right and left orbits to the occipital part of the calvaria. The temporalis muscles were subperiosteally elevated from the frontal bone and retracted bilaterally (flap). All remaining soft tissue attached to the bone was sharply dissected to expose the site and prepare the frontal bone for implantation. Two semi‐onlay implants were bilaterally placed on the frontal bone of the skull of each sheep. Each implant was fixed to the frontal bone with two self‐drilling 1.5 × 4 mm bone screws (Medicon, art no: 68.93.24 A) one in each opposite fixation points of the implant, as outlined in Figure 1a, D‐E. Following fixation of the implants, the soft tissues were closed with absorbable sutures (Vicryl^®^ 2‐0, Ethicon) and the skin was closed with non‐absorbable sutures (Prolene^®^ 2‐0, Ethicon) and surgical staples. The wounds were disinfected using oxytetracycline (Oxytetrin^®^ spray, MSD).

#### 2.3.3 Post‐operative procedure

Each animal was moved to a recovery area and monitored for recovery until sternal recumbency was achieved. After recovery, each animal was returned to its cage and observed for general health. The sheep were group‐housed to allow social contact under standardized conditions, with controlled room temperature humidity and 12 h light cycle. After the post‐operative period, the sheep were group‐housed in a farm setting (Bergerie de la Combe aux Loups), identified by an individual tag in the ear.

#### 2.3.4 Treatments

An IM injection of buprenorphine (Buprecare^®^, Axience, 0.005 mg/kg) was administered at the end of the surgery day, then daily for 2 days post‐surgery. An anti‐inflammatory drug (flunixin, Meflosyl^®^ Injectable, Zoetis, 2 mg/kg) was administered IM daily for 7 days post‐surgery and antibiotics were given for 3 weeks following surgery: amoxicillin (Duphamox LA^®^, Zoetis, IM, once every 2 days, 15 mg/kg) and enrofloxacin (Baytril^®^ 10%, Bayer Pharma, subcutaneous daily, 5 mg/kg). After approximately 2 weeks following surgery, the surgical sutures and staples were removed. The wounds were disinfected with oxytetracycline (Oxytetrin^®^ spray, MSD) once every 2 days until 2 days after the removal of the surgical sutures and staples. When needed, the local disinfection was replaced/extended with povidone iodine (Vetedine^®^ solution, Vetoquinol).

#### 2.3.5 Euthanasia

At 13 weeks, the animals were weighed, and blood and cerebrospinal fluid were sampled under anesthesia. The sheep were euthanized by an intravenous injection of sodium pentobarbital (Dolethal^®^, Vetoquinol).

#### 2.3.6 Dissection

The regions of the implantation sites were carefully exposed, and macroscopic changes were recorded. After examination, the implantation sites were X‐rayed and fixed in 10% neutral buffered formalin (NBF) for the histology process.

### 2.4 Histology

#### 2.4.1 Preparation

After complete fixation in 10% NBF, the implanted sites (*n* = 12) and non‐implanted samples were dehydrated in alcohol solutions of increasing concentration (50%–99.9%), cleared in xylene and embedded in polymethylmethacrylate. The PMMA‐embedded specimens were sectioned for histologic slide preparation. One longitudinal non‐centered section was prepared for each specimen by a micro‐cutting and grinding technique (Exakt™). The sections were stained with modified Paragon for qualitative and semi‐quantitative analysis.

#### 2.4.2 Analysis

Qualitative and semi‐quantitative histopathologic evaluation was performed with an ordinal system ranging from minimal, slight, moderate, marked up to severe. This system was used to score the local tissue effects and the inflammatory response at the implantation sites and was conducted according to the standard (ISO 10993 – Part 6) by certified pathologists. Non‐implanted samples served as references for the structural characterization of the CaP.

### 2.5 Micro‐computed tomography

Two samples were scanned with μCT at the time of manufacturing with a Skyscan 1172 at 100 kV voltage, 100 mA current, 0.5 mm Al + Cu filter, average five and 360° scan with a voxel size of 9 μm. Images were reconstructed with NRecon, and visualized with CTVox software.

After the *in vivo* study, the explants were examined with μCT on a Skyscan 1176 at 90 kV voltage, 298 mA current, 0.1 mm Cu filter, average four and 180° scan with a voxel size of 9 μm. Images were reconstructed with NRecon and visualized with CTVox software. Two different volumes of interest|volume of interest (VOI) were analyzed: (i) One VOI that covered the empty space, that is, the CaP facing the cavity, the cavity itself and the recipient bone facing the cavity (VOI‐1); and (ii) a VOI that covered the cavity without CaP and recipient bone (VOI‐2) (Figure 4a). Software CTAn was employed for analysis while CTVox was applied for bone imaging. The global threshold was determined for newly formed bone (NFB), and soft tissue was extracted as the volume with the thresholds between air and NFB. The NFB threshold was determined from the mean value of individual Otsu‐thresholding. All μCT equipment and programs were from Bruker MicroCT, Kontich Belgium.

### 2.6 Statistical evaluation

All values are presented with the median and range of minimum and maximum values. The results were evaluated by the non‐parametric paired Wilcoxon signed‐rank test with software Prism 9.0 (Graph Pad Software Inc.). The level of significance was set to *p* < 0.05.

## 3 RESULTS

### 3.1 Material characterization

**FIGURE 2:**
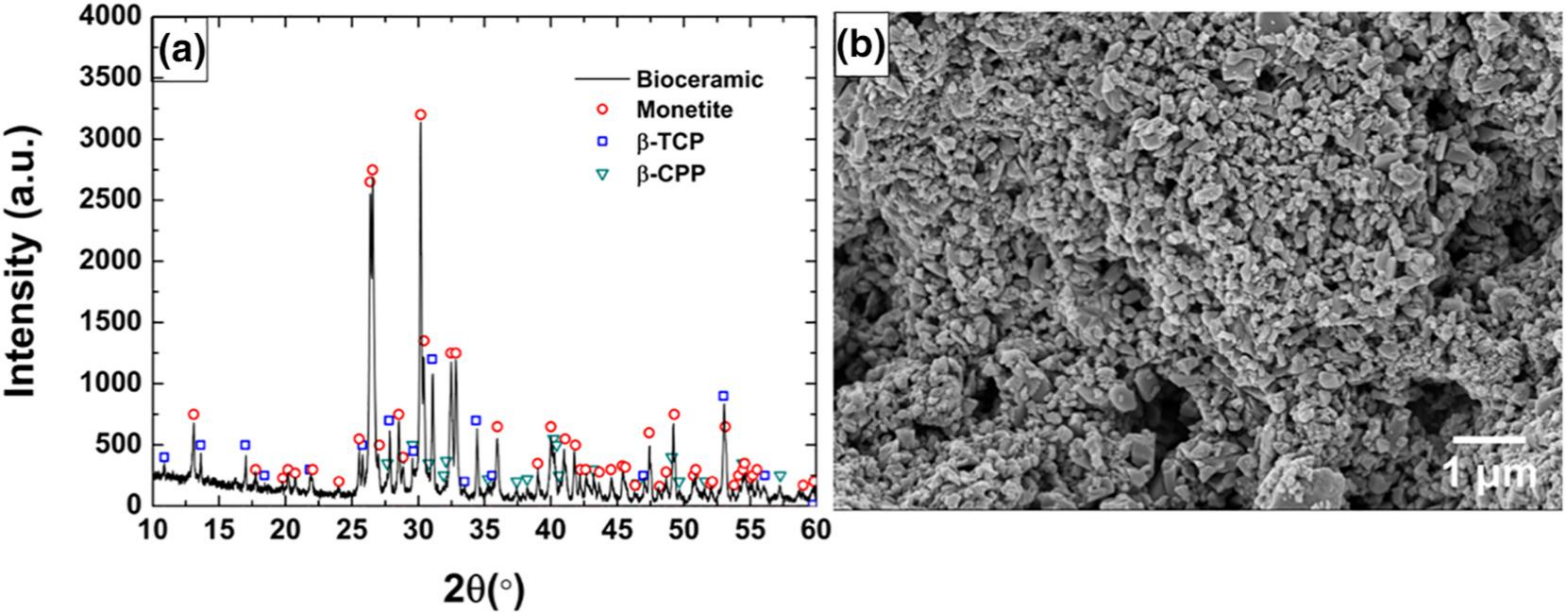
(a) x‐ray diffraction (XRD) pattern of the calcium phosphate (CaP), including phase identification for each peak. (b) SEM micrograph of the bottom CaP component of the implant facing the recipient's bone

The phase composition of the CaP is a mixture of monetite (median 81%, range: 78–85), β‐TCP (median 13%, range: 10–15) and CPP (median 6%, range: 5–6). A representative XRD pattern with the identification of the peaks is depicted in Figure 2a. A SEM micrograph illustrates the morphology of the ceramic implant on the bottom part facing the recipient bone (Figure 2b).

### 3.2 Wettability

**FIGURE 3:**
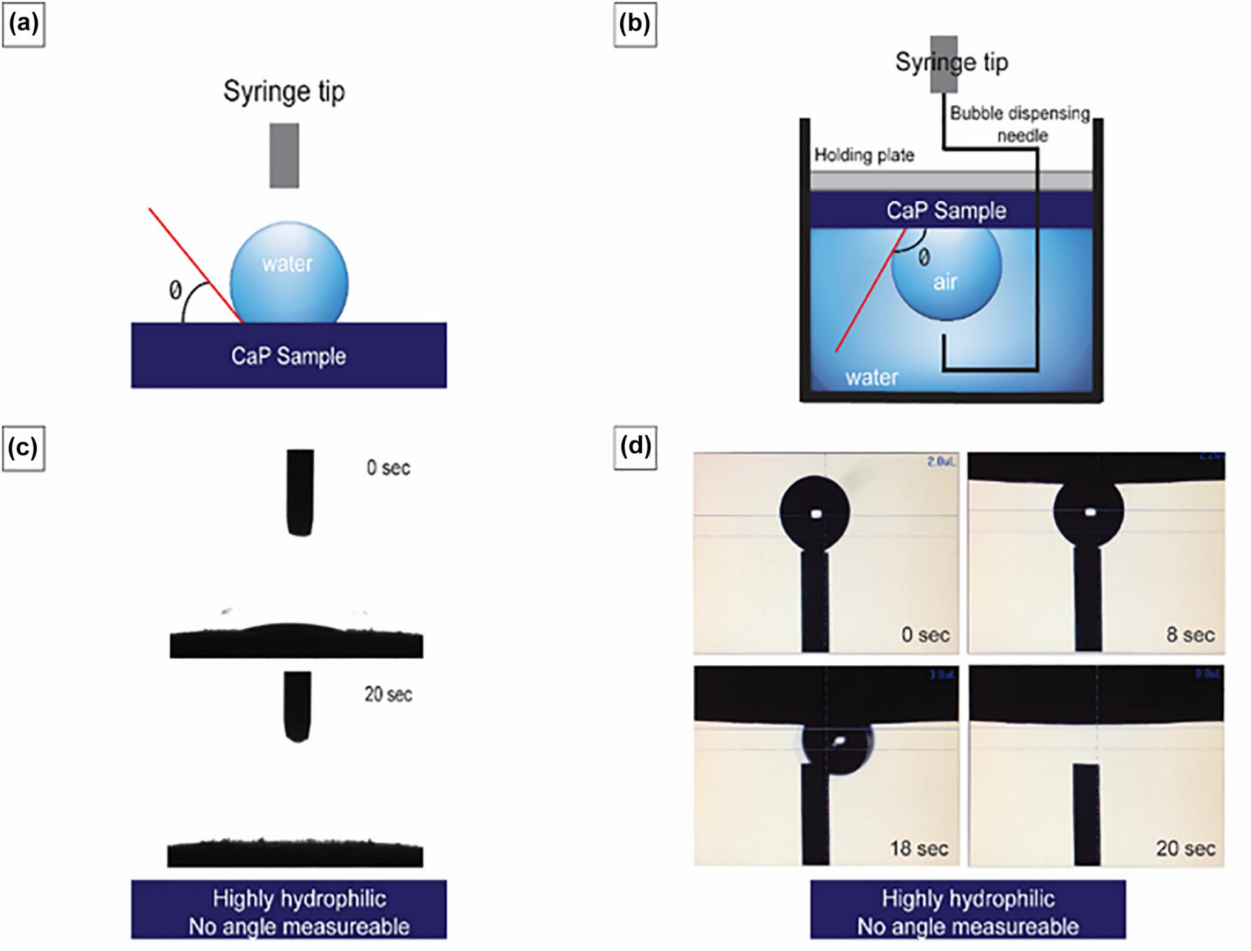
(a) Wettability of calcium phosphate material using the sessile drop and (b) captive bubble methods. Optical images of sessile drop (c) and captive bubble evolution (d) during the time‐course experiment (20 s). θ, contact angle

Calcium phosphate wettability was measured using two methods: sessile water drop and captive air bubble (Figure 3a and b, respectively). Using the former method, a very small contact angle can be observed right after dispensing the drop on the cement surface (Figure 3c, 0 s, Supporting Information Video 1); the sessile water drop was continuously and fully absorbed by the cement in about 1–2 s, so that measurements of the contact angles were not possible due to the very low contact angle and quick absorbance of the water (Figure 3c). Similarly, it was not possible to measure the contact angle using the captive bubble method, since the air bubble did not stably displace water on the surface but quickly escaped from it (Figure 3d, Supporting Information Video 2). The combined results with these two methods reflect the wettability response of a highly hydrophilic material.

### 3.3 Surgery and euthanasia

All the implants were successfully implanted on the frontal bone with no visible abnormalities such as erythema, edema, swelling or signs of infection at any of the implantation sites at the time of dissection. The animals showed no significant weight loss compared to before the surgery. One animal did accidentally get the skin perforated. The lesions were closed on the inner face with absorbable sutures (Vicryl^®^ 4‐0, Ethicon on the right side and Vicryl^®^ 5‐0, Ethicon on the left side). This perforation did not have any impact on the study results since there were neither macroscopic nor microscopic abnormalities.

### 3.4 Histology

**FIGURE 4:**
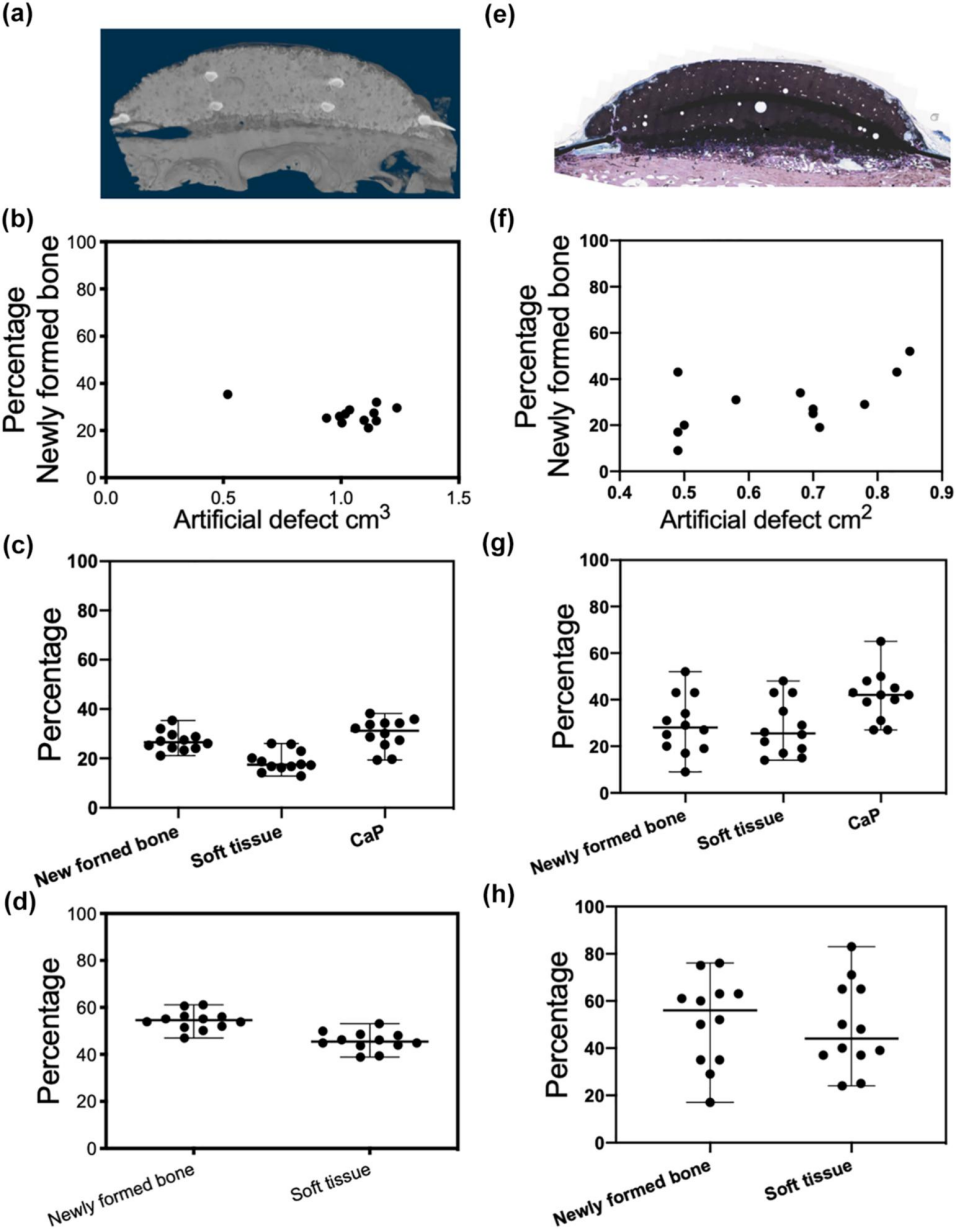
Percentage of tissue ingrowth assessed by μCT (a–d) and histomorphometry (e‐h) after 13 weeks of implantation in a cranial semi‐onlay ovine model. (a) Representative μCT image. (b) There was no correlation between the size of the cavity and the bone volume. (c) The percentage of implant (calcium phosphate (CaP)), newly formed bone (NFB), and soft tissue in the cavity. (d) The percentage of NFB and soft tissue filling the cavity between the CaP and the recipient bone. (e) Histological section of the implants was assessed for histomorphometry. (f) There was no correlation between the size of cavity and the bone area. (g) The percentage of implant (CaP), NFB and soft tissue in the histological section of the cavity. (h) The percentage of NFB and soft tissue filling the cavity between the CaP and the recipient bone. Each individual implant is presented as a dot with the median and range of minimum and maximum values

**FIGURE 5:**
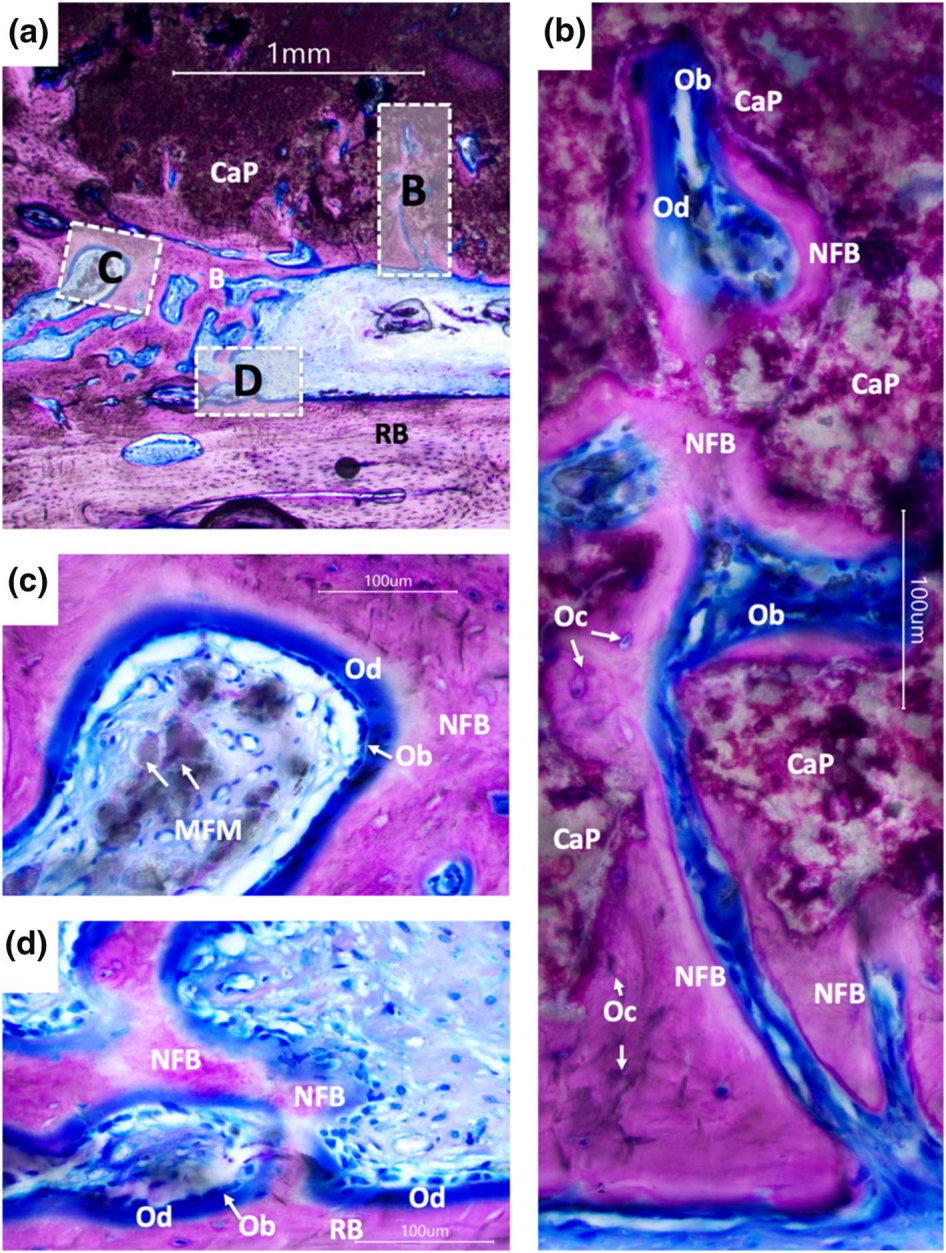
Histological assessment after 13 weeks of implantation in cranial semi‐onlay ovine model. (a) An overview of the interspatial cavity with the calcium phosphate (CaP) at the top and the recipient bone at the bottom. (b) Bone tissue branching into the CaP (c) Magnification of a bone multicellular unit with the presence of CaP degrading macrophages and strands of osteoid secreting osteoblasts lining the CaP and adjacent macrophages. (d) CaP is degraded by macrophages and material‐filled macrophages. Acronyms: CaP, Calcium phosphate; MFM, Material filled macrophages; NFB, Newly formed bone; Ob, Osteoblast; Oc, Osteocyte; Od, Osteoid; RB, Recipient bone

**FIGURE 6:**
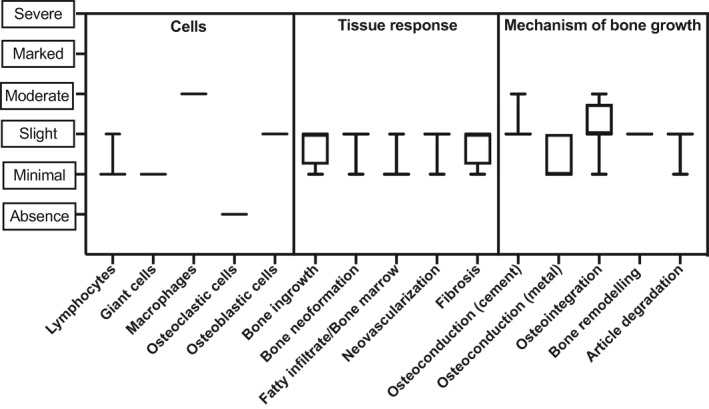
Histopathological evaluation after 13 weeks of implantation in a cranial semi‐onlay ovine model. A moderate rate of macrophages can be seen in the tissue sample with a slight amount of osteoblast, bone ingrowth, bone neoformation and osteoconduction. There is a slight formation of fibrous tissue formation, neovascularization and osteointegration. Each dot represents the median and error bars depict the range of minimum and maximum values

**FIGURE 7:**
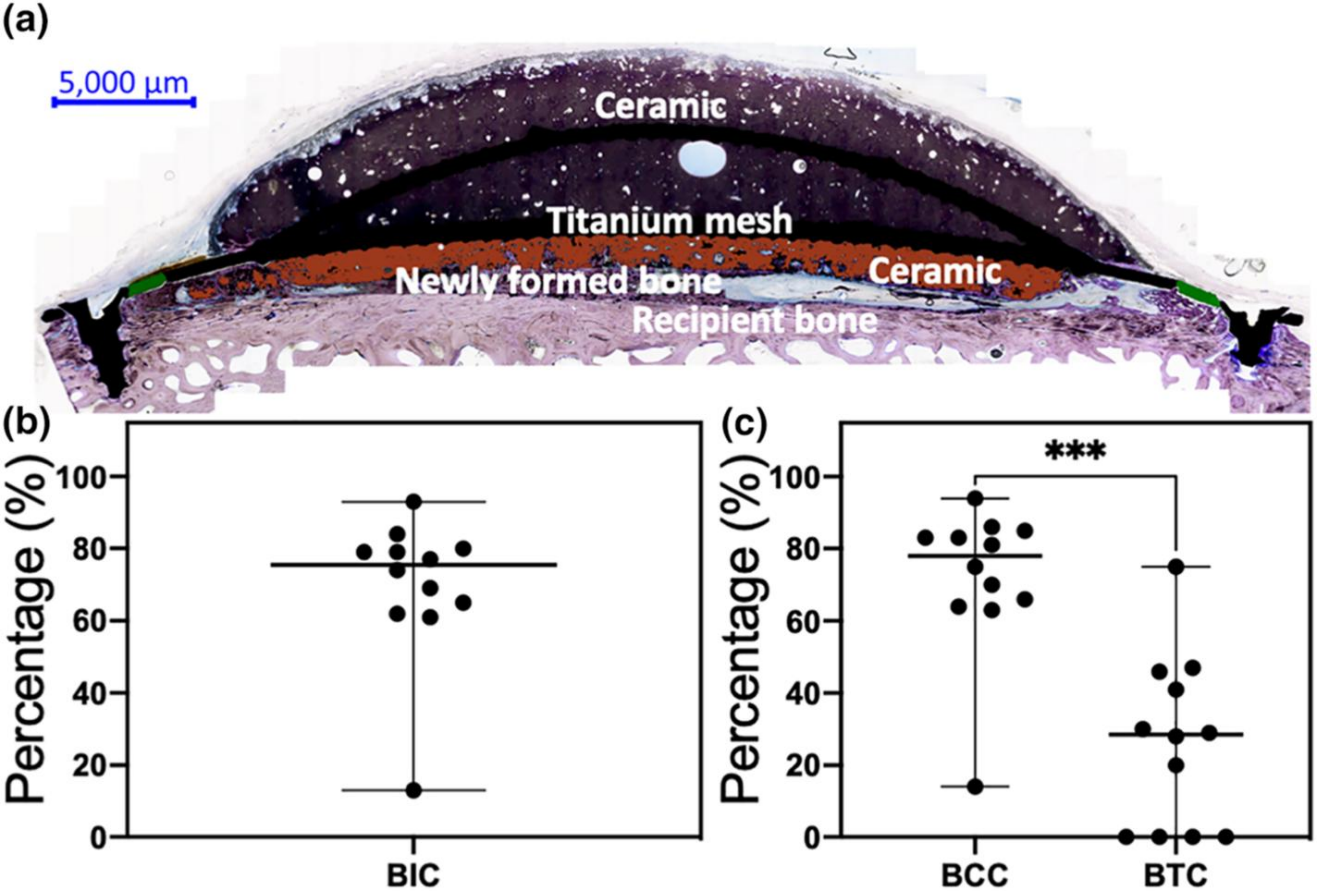
Histomorphometric assessment after 13 weeks of implantation in a cranial semi‐onlay ovine model. (a) A representative overview of the tissue cross‐section used for histomorphometric analysis, depicting the calcium phosphate, titanium mesh, recipient and newly formed bone (NFB). The ceramic evaluated in the bone‐ceramic contact (BCC) measurement and the titanium assessed in the bone‐titanium contact (BTC) are marked in red and green, respectively. (b) There is a 76% bone‐implant contact (BIC) (median: 76%, range: 13%–93%). (c) 78% of the BIC constitutes of BCC (median: 78%, range: 14%–94%) and 29% BTC (median: 29%, range: 0%–75%). Non‐parametric *t*‐test; Wilcoxon matched‐pairs signed‐rank test, ***p < 0.001. Each individual implant is presented as a dot with the median and, range of minimum and maximum values

After 13 weeks of implantation, NFB fills the space between the cranium and the implant, joining them together (Figures 4, 5, 6, 7). Newly formed bone is seen across the whole region of interest with bone colonizing the implant by both ingrowth and direct apposition onto the CaP (Figure 4e–h, 5 and 6).

Histological sections of the implants were assessed for histomorphometry. The interspatial cavity has a median of 28% bone (range 9%–52%), 42% CaP (range 27%–38%), and 26% soft tissue (range 14%–48%) including connective and adipose tissue. Bone tissue fills the cavity between the implant and the recipient bone with a median of 56% bone (range 17%–59%) and 44% soft tissue (range 24%–59%) (Figure 4e–h), which includes connective and adipose tissue. High cell activity is seen by the moderate grade of biodegradation of the implants (Figure 6). This is pronounced around the whole implant surface with material‐filled macrophages present at both the cutaneous and cranial sides (Figures 5c and 6). The material‐filled macrophages observed exhibit a single nucleus, a typical morphological feature of macrophages compared to the multinucleated osteoclasts or giant cells.

There is no sign of a sustained inflammatory reaction, with mainly the macrophagic infiltrate associated with the active biodegradation of the CaP (Figure 6). Osteoclastic cells are absent in the CaP but found on the newly formed. Active bone modeling with osteoblasts (slight grade, Figure 6) is seen in all sites.

The bone‐implant contact (BIC) shows a median of 76% (range 13%–93%). By separating the BIC into bone contact with either the CaP or the titanium part of the implant, a significantly higher amount of bone (*P* < 0.001) is seen in contact with the CaP where the bone‐ceramic contact was 78% (range 14%–94%) in contrast to the bone‐titanium contact, which was 29% (range 0%–75%) as displayed in Figure 7.

### 3.5 Micro‐computed tomography

**FIGURE 8:**
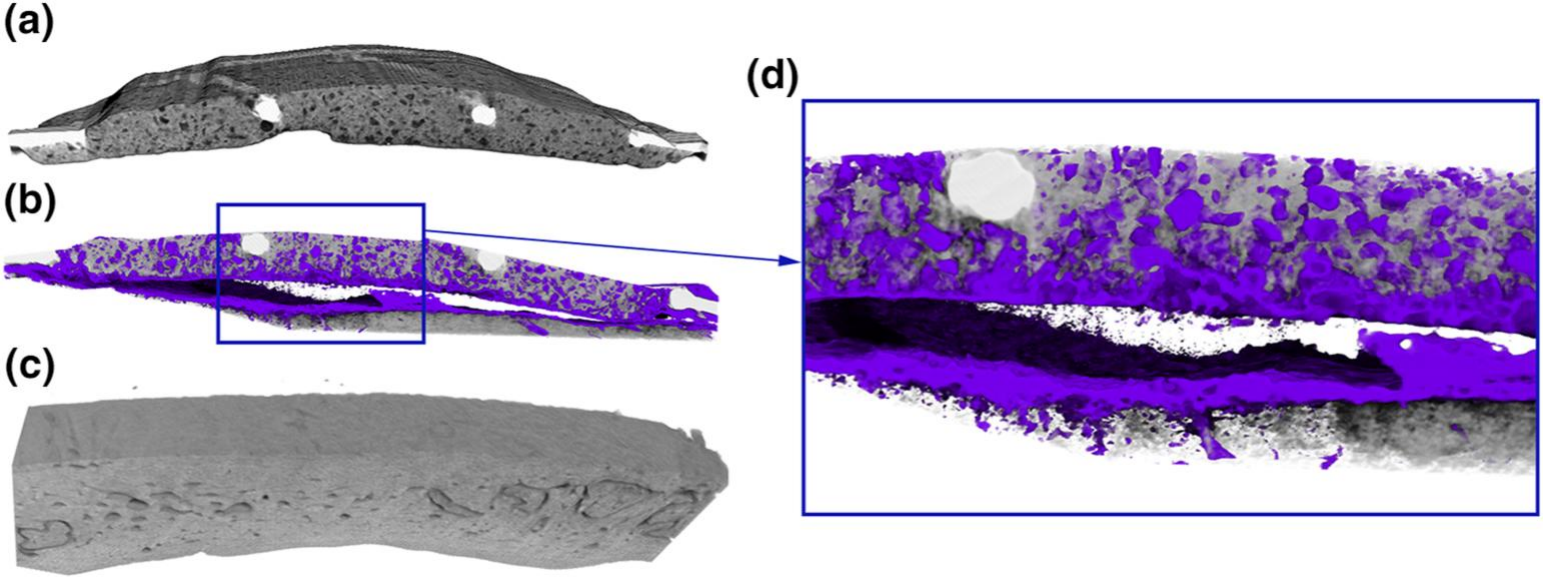
Micro‐computed tomography 3D rendered images after 13 weeks of implantation in a cranial semi‐onlay ovine model. The μCT images depict the network of newly formed bone (NFB) infiltrating the cavity and implant. (a) The part of the calcium phosphate implant facing the bone, prior to implantation. (b) VOI‐1 showing the cavity with the underlying recipient bone and the implant above. Bone tissue infiltrates the implant as well as bone forming from the recipient bone (new bone in lilac). (c) The adjacent recipient bone that was used as a negative control and pre‐treated as the implanted bone, shows no evidence of NFB. (d) A magnification of the cavity showing the NFB (lilac) both expanding from the recipient bone and branching through the implant

The VOI‐1 has a median volume of 1.07 cm^3^ with a range of 0.52–1.24 cm^3^. The space was filled with tissue comprising a median of 27% bone (range 19%–41%), 19% soft tissue (range 7%–32%) including adipose and connective tissue, and 30% CaP (range 10%–44%). The empty space at implantation between the recipient bone and implant (VOI‐2), with a median volume of 0.49 cm^3^ with a range of 0.33–0.61 cm^3^, shows infiltration of tissue with a median of 53% bone (range 46%–61%) and 46% soft tissue (range 39%–54%). In Figure 8, 3D rendered images depict bone‐forming into the porous structure of the CaP, infiltrating the cavity and expanding from the recipient bone (Figure 8d).

## 4 DISCUSSION

In our experiment, a fully synthetic CaP—without the addition of endogenous cells, tissues, osteogenic factors, or decortification—guides vertical bone formation with none to minimal direct contact with the underlying bone. New bone is formed both from the recipient bone and by bone apposition on the CaP. The cavity (median 0.49 cm^3^) is filled with a majority of bone (median 53%) after 13 weeks. A gradient of bone maturity, indicated by the presence of adipose tissue, is documented with the most mature bone found from the side of recipient's bone. No bone growth was observed in the bone area adjacent to the implants sites, which was used as a negative control.

Similar to the report by Bauer and Muschler (Bauer & Muschler, 2000), evidence from the progress of implant incorporation can be found in the current study. In the initial stage following implantation, the hydrophilicity of the CaP may contribute to the creation of a post‐surgical hematoma that soaks the surface of the implant with blood and extracellular fluid. The hematoma formation is a vital component for bone formation with its rich niche of growth factors and cytokines essential to allow cell recruitment and cell migration into the site (Schell et al., 2017). Additionally, a temporary local hypoxic microenvironment should be induced, due to the confined space of the cavity (Huang et al., 2020; Wan et al., 2010), which allows osteoblast and macrophages to colonize the region as they are among the few cells that tolerate such a hypoxic environment (Schell et al., 2017). Following the initial inflammatory stage, fibrovascular tissue is formed and clearly visible in the histological sections, where loose connective tissue is seen in conjunction with areas of NFB, facilitating cell recruitment and acting as a scaffold during the material replacement by bone.

At 13 weeks, macrophages are clearly seen in the histology samples surrounding the CaP of the implant in close vicinity to strands of active osteoblasts. The interplay between macrophages and osteoblasts is crucial for bone formation and the macrophage response dictates the fate of implanted biomaterials (Chang et al., 2008; Loi et al., 2016; Miron & Bosshardt, 2016; Pajarinen et al., 2019; Sridharan et al., 2015; Vasconcelos et al., 2019), which was demonstrated in a previous study (Chang et al., 2008), where the depletion of osteal macrophages inhibited osteoblast differentiation and mineralization. Other studies investigating same CaP composition in non‐load bearing applications show macrophages as the main cells after few weeks, months and years of implantation *in vivo* models (Gallinetti et al., 2021; Malmberg et al., 2021). However, these studies investigate bone formation in calvarial defect models rather than the herein used calvarial model for vertical bone augmentation.

As opposed to the typical osteoclast‐driven material resorption, our results indicate that the CaP replacement is driven by macrophages over time, emphasized by material‐filled macrophages found in close vicinity to the CaP with only a small number of single osteoclasts found actively remodeling the NFB. No osteoclast could be found actively resorbing the CaP that had not yet remodeled to bone further indicating that osteoclasts are not the key players for the resorption of this CaP composition.

Our findings suggest that the placement of the bone‐like CaP material to create a space between the implant and recipient bone does, in fact, create a cavity that is identified as the body's own. The environment created in the space between the recipient bone and implant is likely saturated with calcium ions and inorganic phosphate due to the known passive dissolution of the adjacent CaP, and therefore stimulating bone formation (Bohner & Miron, 2019). Furthermore, the hydrophilic nature of the CaP increases the adhesion and proliferation of osteoblasts (Anselme, 2000; Aronov et al., 2006; Jeong et al., 2019) as can be seen in the long strands of osteoblasts in the histology sections. Interestingly, the individual variation in the interspatial cavity dimensions (VOI‐1; median 1.07 cm^3^, range 0.52–1.24 cm^3^; VOI‐2; 0.49 cm^3^, range 0.33–0.61 cm^3^) does not affect the percentage of NFB.

The void constructed by the CaP implant appears to create a localized milieu ideally suited for bone formation (Ripamonti, 2009; Ripamonti et al., 2011). Even though this approach shares some similarities to guided tissue regeneration techniques in dental applications, our approach allows bone to form without introducing a bony injury or creating a physical barrier to the surrounding soft tissue (Hämmerle & Karring, 1998; Karring et al., 1993; Omar et al., 2019; Stavropoulos et al., 2005).

The CaP is similar to the inorganic part of the bone and the unique combination of mineral phases seems to allow cells to degrade and remodel the CaP into bone in a controlled manner. The evidence of accelerated bone formation by the substantial volume that is populated by the host suggests that the CaP implant itself is recognized as an endogenous tissue. Additionally, the pyrophosphate content of the CaP implant could have a positive biological effect as previous results have shown that initial exposure of pyrophosphate to non‐mineralizing osteoblast initiates mineralization *in vitro* (Kim et al., 2010). Even though pyrophosphate is known as a key regulator in biomineralization *in vivo*, inhibiting spontaneous mineralization, acting as the body's own softener and being part of normal metabolism (Fleisch & Bisaz, 1962; Millán, 2013; Orriss et al., 2016), we hypothesize that the complex crosstalk and feedback loops involved with pyrophosphate in the process of biomineralization could be central to the mechanism of which our CaP material is recognized and replaced by bone. This is supported by the presence of pyrophosphate intracellularly in macrophages exposed to the same CaP material surrounded by newly formed/mature bone, which was one of the key findings from a clinical implant explanted after 31 months due to tumor recurrence (Malmberg et al., 2021). Furthermore, exclusion of the pyrophosphate seems to evoked premature resorption of monetite and β‐TCP of a similar CaP material in large animal studies (Kihlström Burenstam Linder et al., 2019), highlighting the potential promoting role of pyrophosphate in biomineralization (Le Gars Santoni et al., 2022).

A limitation of the present study is the absence of an implant control which would allow for a comparison of the amount of bone and soft tissue formed due to various materials. However, the aim of this study was to investigate the ability of the titanium reinforced semi‐onlay CaP implant to promote vertical bone formation rather than to compare functionality or mechanistical differences between other available materials. In this study, the bone adjacent to the implant served as negative control to verify that bone augmentation did only occur due to the presence of the implant.

Future studies should aim at investigating the importance of the size of the cavity and implant materials to further elucidate the mechanisms and factors behind the successful concept of an artificial cavity. Moreover, engineered interconnected macroporosity could be introduced in order to study how it would affect bone formation and implant colonization. Furthermore, the inclusion of techniques such as immunohistology or gene expression should be incorporate to further study the underlying mechanism of action, especially to elucidate the macrophages response to the material and their crosstalk with other cells. Additional time points would also allow for the assessment of the cellular response over time.

In summary, we demonstrate the possibility of triggering vertical bone augmentation without induced bony injury by suspending a semi‐onlay implant over the recipient bone in an ovine calvarial model. The cavity created by the semi‐onlay allows bone to form both from the recipient's bone and by bone apposition on the CaP. This can be facilitated by either osteoconduction or by providing osteoinductive cues or a combination thereof, causing *de novo* bone formation. Furthermore, the resorption of the CaP and the induced bone formation is governed by macrophages rather than by osteoclasts.

## 5 CONCLUSION

In this proof‐of‐concept, we demonstrated that vertical bone formation can be induced by creating an open space between the recipient bone and a titanium‐reinforced ceramic implant in a calvarial augmentation model without decortification. At 13 weeks, the cavity was filled with a majority of bone and noticeable integration of the implant with the recipient's bone was observed.

Utilizing this approach could allow for cast to be assembled to guide bone formation that can be easily implanted, opening new opportunities for bone reconstruction.

## CONFLICT OF INTEREST

Dr. Christopher Illies, Prof. Conrado Aparicio and Prof. Sune Larsson declare no competing interests. Dr. Viviana R Lopes and Dr. Ulrik Birgersson report personal fees from OssDsign both during the conduct of the study and outside the submitted work. Dr. Gry Hulsart Billström, Dr. Jonas Åberg, Dr. Sara Gallinetti, Prof. Håkan Engqvist and Dr. Lars Kihlström Burenstam Linder have consulting agreements with OssDsign, and Prof. Håkan Engqvist sits on the board of directors of OssDsign.

## AUTHOR CONTRIBUTION

**Gry Hulsart Billström:** Data Curation, Formal Analysis, Visualization, Writing ‐ Original draft, Writing ‐ Review & Editing, **Viviana R. Lopes:** Data Curation, Formal Analysis, Visualization, Writing ‐ Original draft, Writing ‐ Review & Editing, **Christopher Illies:** Formal Analysis, Visualization, Writing ‐ Review & Editing, **Sara Gallinetti:** Data Curation, Formal Analysis, Visualization, Writing ‐ Review & Editing, **Jonas Åberg:** Conceptualization, Writing ‐ Review & Editing, **Håkan Engqvist:** Conceptualization, Writing ‐ Review & Editing, **Conrado Aparicio:** Data Curation, Formal Analysis, Visualization, Writing ‐ Review & Editing, **Sune Larsson:** Data Curation, Writing ‐ Review & Editing, **Lars Kihlström Burenstam Linder:** Conceptualization, Formal Analysis, Writing ‐ Original draft, Writing ‐ Review & Editing, **Ulrik Birgersson:** Conceptualization, Data Curation, Formal Analysis, Project administration, Supervision, Visualization, Writing ‐ Original draft, Writing ‐ Review & Editing

## Supporting information

Supporting Information 1

Supporting Information 2

Supporting Information 3

## Data Availability

The data that support the findings of this study are available from the corresponding author upon reasonable request.
