# Supplementary material for: Guiding bone formation using semi‐onlay calcium phosphate implants in an ovine calvarial model
Source: J Tissue Eng Regen Med. 2022 Feb 23;16(5):435–47. doi: 10.1002/term.3288 (PMC9303616; doi:10.1002/term.3288)
Supplement: Supplementary file 1 — Supporting Information 1 [file TERM-16-435-s001.docx]

Supplementary data

**Guiding bone formation using semi-onlay calcium phosphate implants in an ovine calvarial model**

Authors: Gry Hulsart Billström^1^, Viviana R Lopes^1,8^, Christopher Illies^2^, Sara Gallinetti^3,8^, Jonas Åberg^3,8^, Håkan Engqvist^3^, Conrado Aparicio^4^, Sune Larsson^5^, Lars Kihlström Burenstam Linder^6^, Ulrik Birgersson^6,7,8^

Affiliation:

*1 - Department of Medicinal Chemistry, Translational Imaging, Uppsala University, 751 83 Uppsala, Sweden*

*2 - Department of Clinical Pathology, Karolinska University Hospital, Stockholm, Sweden*

*3 - Department of Engineering Sciences, Applied Materials Science Section, Uppsala University, 75121 Uppsala, Sweden*

*4 – Faculty of Odontology, International University of Catalonia, Josep Trueta, 08195 Sant Cugat del Vallés, Barcelona, Spain*

*5 - Department of Surgical Sciences, Orthopaedics, Uppsala University, 751 85 Uppsala, Sweden*

*6 -Department of Clinical Neuroscience, Neurosurgical Section, Karolinska University Hospital, 17176 Stockholm, Sweden*

*7 - Division of Imaging and Technology, Department of Clinical Science, Intervention and Technology, Karolinska Institute, 14152 Huddinge, Sweden*

*8 - OssDsign, 754 50 Uppsala, Sweden*

**Corresponding author: Ulrik Birgersson, Division of Imaging and Technology, Department of Clinical Science, Intervention and Technology, Alfred Nobels Allé 10, Karolinska Institutet, Huddinge, SE, 141 86 Sweden +46(0)708421054*

Content:

- Supplementary video 1 -Sessile drop results
- Supplementary video 2 - Captive bubble results

Supplementary video 1 -Sessile water drop results

Supplementary video 2 - Captive air bubble results
